# Emotional Experiences of Pregnant and Postpartum People with Confirmed or Suspected COVID-19 Infection During the Initial Surge of the Pandemic

**DOI:** 10.1089/whr.2021.0143

**Published:** 2022-04-04

**Authors:** Natalie C. Spach, Elana F. Jaffe, Kristen A. Sullivan, Cindy Feltner, Anne D. Lyerly, Ilona T. Goldfarb

**Affiliations:** ^1^ Center for Bioethics and Department of Social Medicine, University of North Carolina School of Medicine, Chapel Hill, North Carolina, USA.; ^2^ Department of Public Health Leadership, University of North Carolina Gillings School of Global Public Health, Chapel Hill, North Carolina, USA.; ^3^ Department of Obstetrics and Gynecology, Massachusetts General Hospital, Boston, Massachusetts, USA.; ^4^ Department of Obstetrics and Gynecology and Reproductive Biology, Harvard Medical School, Boston, Massachusetts, USA.

**Keywords:** COVID-19, pregnancy, birth, postpartum, women's views, qualitative methods

## Abstract

**Objectives:**

The COVID-19 pandemic may have a unique emotional impact on pregnant people. This qualitative study aimed to characterize the emotional effects of the COVID-19 pandemic on pregnant and recently pregnant patients who had either suspected or confirmed COVID-19 infection during the initial 6 months of the pandemic.

**Methods:**

Pregnant and recently pregnant participants (*n* = 20) from Massachusetts General Hospital Obstetrics and Gynecology clinical sites with suspected or confirmed COVID-19 infection were interviewed about their experiences during the COVID-19 pandemic. Interviews were transcribed and coded using NVivo 12 software. Using data display matrices, thematic analysis was performed to identify emergent, crosscutting themes.

**Results:**

Twenty pregnant and postpartum patients participated of whom 12 had confirmed COVID-19 infection and 8 had suspected infection. The most frequently described emotions were anxiety (90%), uncertainty (80%), fear (70%), relief (65%), and sadness (60%). The following three crosscutting themes were identified: risk, protection, and change. The ways in which participants articulated their emotional reactions to the themes of risk, protection, and change were complex and varied.

**Conclusions:**

There was a broad range of negative and positive emotional experiences of pregnancy, birth, and the postpartum period during the first 4 months of the COVID-19 pandemic. A better understanding of pregnant people's emotional experiences may lead to changes in clinical practice and institutional policies that are more supportive of their needs and congruent with their values.

## Introduction

The COVID-19 pandemic has generated extraordinary fear and loss, which may uniquely affect the emotional wellbeing of pregnant people.^1^ Even in nonpandemic times, the perinatal period can be an emotionally and socially vulnerable time, and confers an increased risk of psychiatric complications.^2^ Pregnant people have an increased risk of severe COVID-19 disease,^3^ which in turn heightens the risk of pregnancy complications, such as preterm birth and preeclampsia.^4,5^ Adverse perinatal outcomes, regardless of the cause, are associated with worse psychological health in the postpartum period.^6^ Thus, COVID-19 infection and associated complications occurring during pregnancy or birth may negatively impact the emotional wellbeing of pregnant and postpartum people.^7^

Given the need to prevent the spread of COVID-19 within hospitals, many infection control policies were implemented at the beginning of the pandemic, which impacted the care environment for laboring and postpartum patients and their families, including restrictions on visitors in delivery rooms, recommendations for neonatal separation, and institution of virtual prenatal and postpartum appointments.^8,9^ Such policies may have significant emotional effects on patients. Recent data show a rising incidence of perinatal mood disorders^10,11^ as well as post-traumatic stress disorder among women who delivered during the pandemic.^12^

Much of the literature has assessed the general psychological experiences of pregnancy throughout the pandemic.^13^ While one qualitative study from Brazil explored the emotional effects of COVID-19 infection on pregnant people,^14^ there is limited qualitative data on the emotional experiences of U.S. pregnant people with COVID-19 infection.

To address this void, we queried the lived experiences and emotional responses of pregnant and recently pregnant people in the Boston area who had either confirmed or suspected COVID-19 infection during the first wave of the pandemic, a time when Massachusetts was a COVID-19 hotspot,^15^ and when strict infection control policies and social distancing recommendations were widespread in the area.

## Methods

The data for this study, “Experiences of Pregnancy and Birth Among Women Impacted by COVID-19,” were obtained between March 2020 and August 2020. Qualitative methodology was employed to surface and explore the range of themes, views, and experiences of pregnant people. Thematic analysis was used to identify emergent patterns within and across participants.^16^ This study was approved by the Massachusetts General Hospital Institutional Review Board.

### Study population

Since March 2020, all pregnant and postpartum patients cared for by the obstetrical service at Massachusetts General Hospital who were suspected or confirmed to have COVID-19 based on exposures, symptoms, or test results were tracked on a clinical database. Patients on this list were eligible to be approached for participation if they met the following inclusion criteria: pregnant or recently pregnant, 18–50 years of age, symptomatic for COVID-19 with either a positive, negative, unknown test result, or asymptomatic with positive COVID-19 test.

Patients were approached and offered participation in the study during a scheduled prenatal or postpartum care visit (either virtual or in-person) by clinic staff. Willing participants were contacted virtually by a research assistant and consented. This process was approved by the Massachusetts General Hospital Institutional Review Board.

### Process and data collection

We conducted in-depth phone interviews in English with pregnant and recently pregnant participants with suspected or confirmed COVID-19 infection to explore pregnancy, birth, and postpartum experiences during the COVID-19 pandemic. Qualitative methods^17^ were employed to capture a broad range of themes. Verbal informed consent was obtained from all participants before interviews, which was approved by the IRB due to pandemic circumstances and need for physical distancing.

**FIG. 1.**
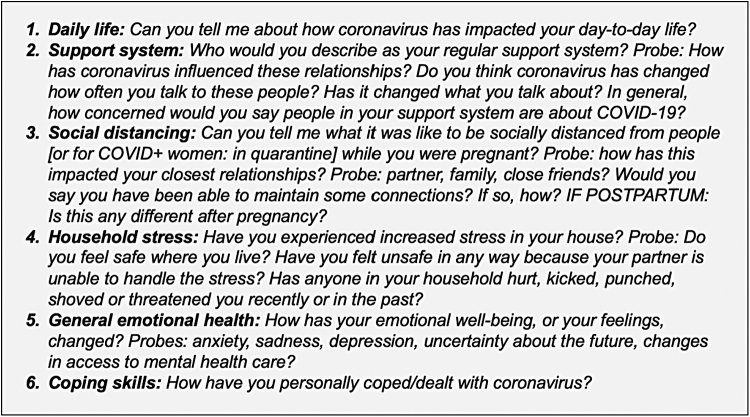
Interview questions on psychosocial and emotional impact of COVID-19.

Using a semistructured interview guide, we asked participants questions about their experiences of pregnancy, birth, and the postpartum period during the COVID-19 pandemic, including questions about their pregnancy history, COVID-19 status and history, health care experiences, and emotional and physical health. We also explored the impact of the COVID-19 on their daily life, support systems, and coping strategies during the pandemic (Fig. 1). Participants provided information about sociodemographic characteristics and reproductive health history. All phone interviews were audiorecorded, transcribed, and uploaded into NVivo 12 software for thematic analysis.^16^

### Data analysis

This analysis focuses on participants' emotional experiences related to navigating the perinatal period in the context of suspected or confirmed COVID-19 infection. We developed codes *a priori*^16^ from the qualitative interview guide and used NVivo software to code transcripts. The coding process was iterative and new codes were added throughout. We double-coded 20% of transcripts to ensure intercoder reliability and resolved coding discrepancies through discussion. Our team-based approach to thematic analysis included discussions around the ways in which researcher assumptions and beliefs were affecting our identification of themes and data interpretation.

Using display matrices, we identified emergent themes related to participants' self-described emotional responses. For each theme, representative quotes were selected and are displayed below by pseudonym, race/ethnicity, pregnancy trimester, and COVID status.

## Results

### Participant sociodemographic characteristics and COVID history

The final sample consisted of 20 pregnant or recently pregnant participants from Massachusetts General Hospital clinical sites. Participant ages ranged from 28 to 49 years (mean age = 35.6 years); 65% (*n* = 13) were non-Hispanic White, 20% (*n* = 4) were non-Hispanic Black, and 15% (*n* = 3) were Hispanic/Latinx (Table 1). At the time of their interviews, 35% (*n* = 7) of participants were in the second trimester, 30% (*n* = 6) were in the third trimester, and 30% (*n* = 6) were postpartum; 60% (*n* = 12) of participants were multigravidas. Most participants, 75% (*n* = 15), were employed, 65% (*n* = 13) had a college degree or higher, 80% (*n* = 16) were privately insured, and 80% (*n* = 16) were married.

**Table 1.** Sociodemographic Characteristics of Participants

Characteristics	*n*	%
Age		
25–34	10	50
35–44	9	45
45+	1	5
Pregnancy trimester		
First trimester	1	5
Second trimester	7	35
Third trimester	6	30
Postpartum	6	30
Gravidity		
Primigravida	8	40
Multigravida	12	60
Race/ethnicity		
Non-Hispanic White	13	65
Non-Hispanic Black	4	20
Hispanic/Latinx	3	15
Education level		
High school/GED	5	25
Associate's degree	2	10
Bachelor's degree	8	40
Master's degree or higher	5	25
Employment status		
>1 Full or part-time job	2	10
1 Full or part-time job	13	65
Unemployed	5	25
Insurance status		
Private	16	80
Medicaid	4	20
Marital status		
Married	16	80
Unmarried	4	20

GED, General Educational Development.

At the time of the interview, 55% (*n* = 11) were COVID positive, 15% (*n* = 3) were “persons under investigation” (PUI) who tested negative, and 30% (*n* = 6) were PUI who were symptomatic for COVID-19 illness but either not tested or had an unknown test result (Table 2).

**Table 2.** COVID-19 Testing and Symptom History of Participants

COVID-19 history	*n*	%
Test status		
Tested positive	11	55
Tested negative	3	15
Not tested/unknown test result	6	30
Symptoms		
Severe	6	30
Moderate	3	15
Mild	8	40
None/asymptomatic	3	15

### Self-described emotional responses among participants

Table 3 shows the number of participants who described specific emotions at least once during the interview. The most common negative emotions described by participants were anxiety (90%), uncertainty (80%), fear (70%), sadness (60%), and disappointment (55%). The most frequently described positive emotions were relief (65%), trust (55%), and feelings of acceptance (35%) (Table 3).

**Table 3.** Participants' Self-Described Emotions During Pandemic

Emotion	*n*	%
Negative		
Guilt	7	35
Fear	14	70
Anxiety	18	90
Depression	6	30
Mistrust	10	50
Disappointment	11	55
Loneliness and isolation	9	45
Stress	11	55
Uncertainty	16	80
Frustration/anger	9	45
Overwhelmed	5	25
Sadness	12	60
Positive		
Hope/optimism	6	30
Calmness	3	15
Trust	11	55
Gratitude	8	4
Relief	13	65
Acceptance	7	35

Emotions described at least once in response to any question from the interview.

### Themes

Three intersecting themes were identified related to participants' emotional experiences of COVID-19: risk, protection, and change. The intersections between these themes highlight the compounding emotional effects of pregnancy and the pandemic.

#### Risk

Many respondents emphasized that pregnancy and COVID-19 represented a time of risk. Participants discussed how the pandemic compounded the sense of risk and exacerbated emotional responses to risk. Overwhelmingly, participants expressed fear and anxiety related to the risks associated with COVID-19 in pregnancy. Many respondents also described fear about the risk of fetal anomalies due to COVID-19. While some respondents were concerned about the immediate risk of anomalies, others worried about the long-term risks from *in utero* infection on their child:

“In ten years, are they going to diagnose my child with something that has some sort of connection with me being pregnant with COVID?. Like developmental delays?”

—Charlotte, White, Third Trimester, COVID Positive

Other participants expressed that the lack of data was a source of anxiety:

“It's been an anxious time. There are too many unknowns right now about how this will affect my baby even though how this will affect my baby… There's just not enough data out there.”

—Jane, White, Second Trimester, COVID Positive

Participants with a history of pregnancy loss were particularly worried about the immediate risks of COVID-19 causing a demise in their current pregnancy:

“I had an intrauterine fetal demise at 23 weeks… I've just had added anxiety just with this pregnancy in general… Coronavirus has just been another obstacle.”

—Keisha, Black, Second Trimester, COVID Positive

Others voiced anxiety about the risk of obstetric-related complications from COVID-19, including preterm labor. As many COVID-positive participants focused on the risks to the fetus and pregnancy, others expressed fear for their own lives:

“I was really worried because people die from this…I was scared. I remember the night I said, “Oh my god am I going to die?”

—Brianna, Black, Third Trimester, COVID Positive

For many participants, making decisions about risk—weighing the risks and benefits of each action—was associated with a variety of emotions. One participant described how decision making heightened her sense of uncertainty and anxiety as a new mother:

“I felt uncertain and unsure about whether I was making the right decisions. I tried to use all the resources available to me… but there is no clear guide to what to do… I definitely felt more anxious… It definitely brought up a lot of uncertainty as a new mom.”

—Sophia, White, Postpartum, PUI not tested

Some participants discussed the stress of decision making and risk evaluation during a time when information sources were confusing or unreliable, expressing mistrust and uncertainty:

“It was really hard for me to trust anything because of the media… There was a lot of misinformation… There was a lot of going back and forth with opinions from people, from scientists, opinions from doctors that—that that conflicted with each other… Every day it was something new… My trust and confidence definitely started to dissipate after a little while.”

—Amelia, White, Postpartum, COVID Positive

#### Protection

Another emergent theme was protection, which led to a range of emotional responses. The notion of protection was raised in a variety of contexts, including an internal impulse toward protection as well as external experiences of protection from health care workers and systems. Many participants voiced their desire to protect their babies, families, health care staff, and others from COVID-19. One respondent who was COVID positive described guilt for not protecting her baby from the infection:

“How did I let this happen to myself and to the baby? I felt very guilty, I felt like I did something wrong… I felt like I was supposed to protect myself and the baby… I made sure to go to stores during pregnancy hours, I made sure to wash my hands and wear a mask, and when I got into the car wiped everything down, I took all the precautions… I'm following all the rules, and then someone else there didn't follow the rules, and now I have it and my baby's in trouble.”

—Charlotte, White, Third Trimester, COVID Positive

Although contracting COVID-19 signified a lack of fetal protection for some participants, others described positive emotions and felt that the infection represented protection:

“I feel lucky in a way because I was sick already. I feel like I'm protected to an extent, so, hopefully the antibodies, and hopefully my child will also have antibodies and will not get sick even if exposed, so in a weird way, it comforts me that I was sick.”

—Isabella, White, Third Trimester, COVID Positive

Participants who were COVID positive cited a range of emotions related to their desire to protect health care professionals from COVID-19 infection:

“I also had a lot of anxiety around exposing people to me. I had a lot of guilt around that and was nervous about going into the emergency room and seeing all the doctors and nurses who were staying healthy taking care of other people. I was anxious about them getting sick because of me.”

—Larissa, White, Postpartum, COVID Positive

While some emphasized their impulse to protect others, others described experiences of receiving protection from external sources, including physicians and health care systems.

“My OB office and my PCP office, they were calling me like every day checking in… They made me feel confident… When I was at the 7-day, 8-day mark and my symptoms weren't getting worse, I was like, ‘okay this is going to be the worst of it, I'm going to be fine.’”

—Charlotte, White, Third Trimester, COVID Positive

For many, frequent contact and outreach from health care providers were a positive form of protection and led to feelings of comfort and support:

“It makes you feel like somebody cares. [It] feels like, “Okay, I'm with you. I just can't see you, but I'm with you.”

—Brianna, Black, Third Trimester, COVID Positive

Some focused specifically on how the patient–physician relationships created a sense of protection, leading to more trust, comfort, and relief:

“I love my doctor… he's the best… I was already comfortable with him. I trust him… We have a good relationship and so that I was confident that if anything was wrong, I would tell him, and he would help me.”

—Amelia, White, Postpartum, COVID Positive

Protection also signified infection control measures from health care facilities. Some participants described protective measures with positive emotions, including acceptance, trust, and reassurance, whereas others reported negative emotional experiences:

“On delivery day, I was totally just beside myself. I was devastated because they were telling me I can't do skin to skin with the baby, that I have to wear a mask if I want to breastfeed him, the bassinet has to be six feet away from my bed, and I was just devastated… I was crying… That first time that you hold the baby, it's like—the best thing in the whole world. When they came in and told me like I said that I was positive for the coronavirus I was totally devastated… I just felt like my rights were being violated. I felt like they couldn't tell me that I couldn't hold my own kid…I was angry… I was hurt.”

—Amelia, White, Postpartum, COVID Positive

#### Change

Another salient theme was change, which was evoked in a variety of contexts, including changes in prenatal care, birth, and postpartum care, as well as broader changes in social structures and daily routines. The ways in which participants responded emotionally to change were varied and complex. For many participants, changes in typical prenatal care and birthing experiences were associated with anxiety and uncertainty. Participants who had less frequent appointments or telemedicine visits voiced heightened anxiety:

“It's really frustrating and caused me extreme anxiety… I had two miscarriages. And both during the first trimester, and one I didn't know that there was no heartbeat. And I didn't have a doctor's appointment for a while. So, this [appointment] was to make sure that I didn't miscarry.”

—Madison, White, First Trimester, PUI Not Tested

Others expressed grief related to the changed policies for support personnel allowed at prenatal appointments:

“I would love to take my girls so they can see the baby. I hate that. And I hate that my husband, he can't go in with me. I would like for us to go as a family… It's sad that I have to be there by myself. I would like to experience that happiness with my family. They're so excited about it and they can't see the baby on the ultrasound; it makes me sad.”

—Viola, Black, Second Trimester, PUI COVID Negative

While some respondents felt sadness and grief related to the changed health care experiences, others voiced acceptance and understanding:

“I think it's like a really special moment for my kids to meet their sibling, but it's just hard, not being able to. I understand it's because of the virus and I want to keep them safe.”

—Jocelyn, Latina, Third Trimester, COVID Positive

Another participant responded to the changes in the birthing experience with gratitude:

“Labor and the c-section were so traumatic…not to be able to be close to my daughter for a little bit was even more traumatic… but I'm just so happy that I have a healthy daughter… that's all I think about… I'm very grateful for that.”

—Christine, White, Postpartum, PUI COVID Negative

For some participants, broader social changes from the pandemic transformed social experiences of pregnancy:

“Coronavirus has changed everything. We're not able to have a baby shower, which we were hoping to have in June.”

—Keisha, Black, Second Trimester, COVID Positive

Some respondents endorsed grief associated with these changed social experiences of pregnancy and birth during the pandemic:

“I was mourning… It felt a little bit like we got robbed of the experience that we hoped to have with a new baby.”

—Sophia, White, Postpartum, PUI Not Tested

For many participants, there were changes in social structures, which led to isolation, loneliness, depression, and even guilt:

“I still feel guilty now for my kids because I feel like they're missing out on a lot of experiences that they really need to experience. There is a lot of ‘mom guilt.’”

—Amelia, White, Postpartum, COVID Positive

While some participants described the changes as “depressing,” others voiced more positive emotional responses. One participant reported gratitude associated with the changed daily routines:

“I'm almost grateful for having had to be stuck at home, leading up to having this baby because I was happy to have a lot of one-on-one time with our firstborn before this one came along. I feel like I cherish those times.”

—Larissa, White, Postpartum, COVID Positive

Overall, participants voiced mixed and complex emotional responses to the health care and broader social changes from the pandemic.

## Discussion

Among our sample of pregnant participants with suspected or confirmed COVID infection in a COVID hotspot during the first surge of the pandemic, we found the most frequently described emotions were anxiety, uncertainty, fear, and relief, which is consistent with existing literature.^18,19^ Our findings underscore the sense of threat,^20^ danger, and crisis felt by pregnant people during the initial surge of the pandemic. These emotions—and their associations with risk, protection, and change—converge at the intersection of pregnancy and COVID-19. While some experiences related broadly to navigating the perinatal period during COVID-19 generally (and may apply to a broad range of pregnant people), others are specific to being diagnosed with COVID-19 in pregnancy.

Although risk in pregnancy is often evoked in a context of weighing the risks of a medical intervention,^21^ its significance is broader here and pertains to both pregnancy-specific and pandemic-related risks. Our data suggest that the heightened sense of risk from the pandemic may compound the emotional stress and anxiety about pregnancy-related risks and vice versa.

Unclear communication due to the lack of data surrounding risks of COVID infection in pregnancy may have contributed to worse emotional experiences, including anxiety, mistrust, and uncertainty. Common risk distortions in pregnancy may have been compounded by the surge of misinformation in the pandemic.^22^ Timely and clear communication of public health messages about risks for pregnant people may help not only to combat misinformation^23^ but also to alleviate emotional stress in future crises.

Our findings reveal that unknown risks were an important source of emotional distress for pregnant patients, which has been reported in other studies assessing COVID-negative pregnant populations.^18^ Our findings further demonstrate how “fear of the unknown” manifests as fear about the unknown risks of perinatal infection to the fetus, underscoring the need for data collection, research, and surveillance in pregnant populations, and clear communication of risks to patients who acquired COVID during their pregnancies.

Rigorous data collection and perinatal surveillance systems, such as UKOSS,^24^ are particularly crucial going forward, given that during the first few months of the pandemic, the data on the risk of severe COVID-19 disease in pregnancy was slow to emerge because the pregnancy status was unknown for over 70% of COVID-19-confirmed cases among women of reproductive age.^25^

In addition to risk, participants expressed their desire to operate as agents of protection, keeping not only their babies, but also their families and health care workers safe from COVID-19. While notions of protection in pregnancy are typically discussed in contexts of maternal protective behaviors and antenatal attachment to the fetus, our findings highlight the ways pregnant people's protective impulses extended beyond the fetus, for instance to family members, health care providers, and their community. Emerging literature has shown increased altruism, particularly among people exposed to COVID-19^26^ throughout the pandemic, and our data suggest that pregnant people are no exception.

Our findings also highlight experiences—positive and negative—of being the object of another's protection. They suggest that trusting relationships with health care providers are central to pregnant people's positive emotional experiences with protection, which is consistent with other literature showing that pregnant people who had frequent contact with providers during the pandemic had lower stress levels^18,27^ and rates of postpartum depression.^28^ While certain protective measures were emotionally comforting to participants, others, including infection control policies, had negative emotional effects on pregnant people. Other studies have documented pregnant people's negative psychological experiences with protective measures such as neonatal separation.^29^

Our findings add to this literature and show how many protection measures, even if well intentioned, were not congruent with the priorities of pregnant people and may have caused emotional and psychological harm. In future pandemics, the short- and long-term psychosocial consequences of infection control measures must be considered and should have as strong an evidence base as possible, given their outsize impact in pregnancy.

As participants discussed how protective pandemic measures altered experiences of pregnancy and birth, change emerged as a salient theme. Pregnancy represents a time of transformation, and the pandemic has been filled with unprecedented social and economic change. The two intersecting experiences of pregnancy and COVID may have compounded emotional responses to change.

To many respondents, the disrupted health care and social experiences of pregnancy, birth, and parenthood in the context of COVID-19 were associated with negative emotional responses, which has important implications given that prenatal maternal stress due to disrupted perinatal care during COVID-19 has been linked with adverse perinatal outcomes.^30,31^ To many, health care and social disruptions represented a loss associated with grief, sadness, and guilt.

These emotions are not unique to the COVID–pregnancy context, as many women have previously reported grief when births do not go as planned.^32^ Prior research has suggested that women's positive or negative framing of their birth narratives are more related to control and autonomy than to the actual events of the birth.^32^ Thus, promoting pregnant people's choice—optimizing their feelings of control when possible—may be an important approach to help mitigate the emotional effects of altered pregnancy and birthing experiences in the pandemic.

In particular, the pandemic-related disruptions in social support systems and isolation negatively impacted participants' emotional experiences. Pregnancy and birth are not only medical, but also psychosocial and emotional experiences that occur within broader social networks.^33^ In general, social support plays an important role during pregnancy, delivery, and the postpartum period, and is associated with improved perinatal mental health outcomes.^34,35^ Throughout the COVID-19 pandemic, increased social support has been shown to play a particularly important role in protecting against depression and anxiety,^36^ especially for pregnant populations.^37^

Given that disruptions in social support networks, especially for pregnant people who contracted COVID and were forced to quarantine from immediate family members, may have harmful long-term effects on pregnant and postpartum people's psychological health and wellbeing,^38^ they may benefit from increased psychosocial support, outreach, and perinatal depression screening.^39^

While the changed experiences of pregnancy during the pandemic represented a loss to some participants, others viewed the changes through a lens of gratitude and acceptance, which are both adaptive coping skills for adversity.^40^ Although transitions in life can represent a vulnerable time, they can also be a time of growth and connection. Our data reveal that many participants approached pandemic-associated social changes with a psychological flexibility and adaptability, embracing the positive aspects of the change.

Our study has several strengths. We conducted virtual interviews with COVID-positive patients in real time during the first pandemic surge. While previous studies have sought to broadly describe the changed experiences of pregnancy during the pandemic, our data offer in-depth perspectives on the emotional responses to the illness, infection control policies, and broad pandemic-related social changes of a diverse group of patients with suspected or confirmed COVID-19.

Our study is not without limitations. First, given the sociodemographic characteristics of our study population, these findings are not generalizable to all pregnant populations. Second, our sample was geographically limited to Massachusetts. While our data were obtained from a COVID-19 hotspot during the first wave of the pandemic, future studies assessing different populations in other regions of the United States may raise different considerations.

Third, our data were collected during the first 4 months of the pandemic. At that time, the risks of COVID-19 in pregnancy were unclear and vaccines were not available. In a postvaccine era with more available data on COVID in pregnancy, pregnant people may have new and distinct concerns related to risk. However, our data can serve as a cautionary tale for future pandemics and the impact of early infection control responses.

Our data reveal the extensive emotional impact of the COVID-19 pandemic on pregnant people. Clinical practices and policies should aim to improve access to mental health to support the emotional needs of pregnant and recently pregnant people. Our findings also highlight the complex emotional reactions to various infection control policies and practices from the early pandemic, including infant separation practices and partner support. Although they were well intentioned, some policies had a devastating emotional impact. In future pandemics, a better understanding of pregnant people's perspectives and emotional experiences may lead to more directly supportive and transparent clinical practices and institutional policies that are more aligned with their needs and values.

## Abbreviations Used

GED: General Educational Development
PUI: persons under investigation
